# Decarbonization at What Cost? Assessing the Agricultural Economic Impact of Mitigation Policy: Evidence From Net‐Zero Targets

**DOI:** 10.1002/gch2.70111

**Published:** 2026-05-07

**Authors:** Yu Lai, Zhaopu Liu, Mingen Zhao, Cheng Zhang, Yuchun Zhu

**Affiliations:** ^1^ College of Economics and Management Northwest A&F University Yangling China; ^2^ School of Economics Central University of Finance and Economics Beijing China

**Keywords:** agricultural output value, climate action, mitigation policy, net‐zero targets, no poverty

## Abstract

Typically, the effectiveness of mitigation policy is assessed jointly across environmental, economic, and social dimensions. Agriculture is recognized as the most climate‐sensitive industry and three‐quarters of the global poor depend on agriculture for their livelihoods. Hence, employing net‐zero targets for 143 nations as a novel angle, this study designed a multi‐period difference‐in‐differences model to assess this statement, with a particular focus on its agricultural economic impact. Results indicated that mitigation policy is expected to reduce agricultural output value by 7.9% and the magnitude of emission reduction is nearly half that of output reduction. This finding implies that decarbonization is not merely a sacrifice of agricultural output, but may lead to its destruction. Moreover, mitigation policy exhibits a statistically significant negative impact on no poverty. Notably, observed output losses is primarily attributed to stagnant mitigation technology. Fortunately, this study identified that the output‐enhancing effect of adaptation policy can largely compensate for these losses. To achieve a sustainable future, global entities must collectively optimize the deployment of climate action across multiple fronts to practically enhance the effectiveness of mitigation policy.

## Graphical abstract

Decarbonization is not merely a sacrifice of agricultural output, but may lead to its destruction. This study has woven interlinkages among SDG1 (No Poverty), SDG12 (Responsible Consumption and Production), and SDG13 (Climate Action) into an analytical framework. This finding implies that the effectiveness of mitigation policy should be assessed jointly across environmental, economic, and social dimensions.

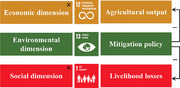

## 1 Introduction

As climate risk has been observed on local, regional, and global scales, climate action constitutes one of the United Nations' sustainable development goals (SDGs). Ripple et al. [1] warned that the inability to effectively mitigate climate change would precipitate the most critical global risk in the foreseeable future. As of the latest count, 216 global entities have implemented a total of 73 625 generalized mitigation policies [2]. Under these circumstances, global decarbonization process had achieved remarkable success and environmental impacts of mitigation policy had been widely documented. For instance, Cheng et al. [3] conservatively estimated that China's carbon emission trading policy reduced CO_2_ emissions by 18% and SO_2_ emissions by 36% in pilot regions. Moreover, Stechemesser et al. [4] scrutinized 1500 mitigation policies implemented between 1998 and 2022 across 41 nations and identified the 63 most successful ones achieved total emission reductions ranging from 0.6 to 1.8 billion metric tons of CO_2_.

Nevertheless, can we take for granted the effectiveness of mitigation policy? Indeed, this question remains a considerable debate. Hoehn [5] argued that conventional benefit estimates are biased due to the presence of substitution and complementarity effects in the valuation of policy impacts, thereby pioneering a multidimensional evaluation framework for environmental policy. Soimu et al. [6] build on this line of reasoning and call for a shift toward more holistic environmental policy assessment. Put simply, the effectiveness of mitigation policy encompasses at least environmental, economic, and social dimensions. Markkanen and Anger‐Kraavi [7] rigorously outlined a range of inequitable impacts of mitigation policy, including health, income, ethnic, and gender inequalities. Unfortunately, their analysis remains at the theoretical level. Hence, an empirical research regarding the agricultural economic impact of mitigation policy constitutes a notable contribution to enriching the understanding of its effectiveness.

Agriculture represents a unique ecosystem, functioning both as a source and a sink of CO_2_. Clark et al. [8] pointed out that even if fossil fuel emissions were eliminated immediately, emissions from the global food system alone would make it impossible to limit warming to 1.5°C and difficult even to realize the 2°C target. It implies that mitigation action in the agricultural sector is urgently needed. Currently, mitigation measures in the agricultural sector are emerging rapidly [9, 10], yet they largely remain in an isolated exploratory stage. Moreover, agricultural economy serves as a vital pillar of national food security, stable economic growth, and harmonious social development. As such, the factors influencing agricultural output had been a perennial subject of interest in both academic and political spheres. Accordingly, positioning our analysis within the agricultural sector is well‐grounded theoretically and of considerable practical importance.

There is an increasing consensus regarding the negative impacts of climate change on agricultural output. For instance, Jarrett and Tackie [11] raised some concerns that climate change are driving increased heat and water stress, which in turn negatively affect agricultural productivity. Meanwhile, coastal aquifers affected by salinity contamination due to rising sea levels are used for irrigation in Tunisia, thereby leading to permanent degradation of several irrigated perimeters [12]. Nevertheless, the impact of climate change mitigation policy on agricultural output remains underexplored. Applying an integrated partial equilibrium modelling framework, Frank et al. [13] found that reducing agricultural greenhouse gas emissions is unachievable without compromising food security. Hence, global mitigation targets within the agricultural sector may come at the cost of food availability. Currently, this contention is based only on predictive data, we address this gap in this paper by incorporating historical data.

In light of this context, our research question can be framed as follows: Does global decarbonization come at the expense of agricultural output? Drawing on social‐ecological systems theory advanced by 2009 Nobel laureate Elinor Ostrom, our research question constitutes a paradigmatic scientific problem. Given the negative evidence emerging from other sectors, we approach this question with a pessimistic expectation. For instance, using a dynamic stochastic general equilibrium model, Lei et al. [14] found that carbon tax reduced manufacturing output in the short term. Naturally, several theories lend support to a favorable answer, with the Porter hypothesis being a prime example [15]. Indeed, the subsequent mechanism analysis is conducted in line with this theory, even though it may ultimately be proven invalid.

Typically, net‐zero targets refer to commitments made by countries and organizations to balance the amount of greenhouse gases emitted with the amount removed from the atmosphere. Dolphin et al. [16] insightful points out that net‐zero targets translate the scientific finding that global warming is determined by cumulative emissions into policy. This argument provides crucial inspiration for designing our difference‐in‐differences (DID) estimation by leveraging net‐zero targets. This novel empirical approach may inform future climate policy evaluation, as existing literature [17] on net‐zero targets largely relies on textual analysis. Besides, mitigation represents a universal challenge confronting all of humanity. However, most mitigation studies remain country‐specific. For instance, Liu et al. [18] contextualized their work within the Chinese context and discovered that mitigation policies interactions delay the achievement of carbon neutrality in China. Wang and Mei [19] conducted an advanced review of mitigation policies in the United States. Against this backdrop, our cross‐country analysis may yield broader, policy‐relevant insights.

Beyond contributing to these abovementioned gaps, more marginal contributions of this paper are as follows. First, guided by endogenous growth theory, we attributed observed agricultural output reductions induced by mitigation policy to a structural contradiction: declining material inputs and stagnant technology supply. This conclusion not only provides a stringent critique of governmental oversight in technological innovation but also identifies the root cause as chronic underfunding of mitigation efforts. Importantly, this granular mechanism analysis pinpoints precise entry points for optimizing governmental decision‐making. Second, against the background of marginalization of adaptation policy, we successfully explored its compensatory effect in offsetting agricultural output reductions induced by mitigation policy. This finding not only offers robust evidence to enhance the priority of adaptation strategy but also underscores the critical need for scholars to integrate both adaptation and mitigation into a unified analytical framework in climate policy research. Lastly, since social impact is likewise a dimension of evaluating the effectiveness of mitigation policy, we placed special emphasis on impoverished groups in the mitigation context and found that losses of critical livelihoods intensify existing poverty conditions, where an intersection between economics and sociology became particularly salient. This insight effectively strengthens our understanding of SDG interlinkages and issues a forward‐looking call for identifying synergistic solutions in a crisis‐prone world, aligning with the stance of the United Nations Department of Economic and Social Affairs (UNDESA).

The structure of this paper is organized as follows: Section 2 provides a detailed account of variable description and model specification. Section 3 primarily presents our empirical findings. Section 4 conducts further discussion and delineates certain limitations. Section 5 concludes all key insights.

## 2 Methodology

### 2.1 Variable Description

**FIGURE 1:**
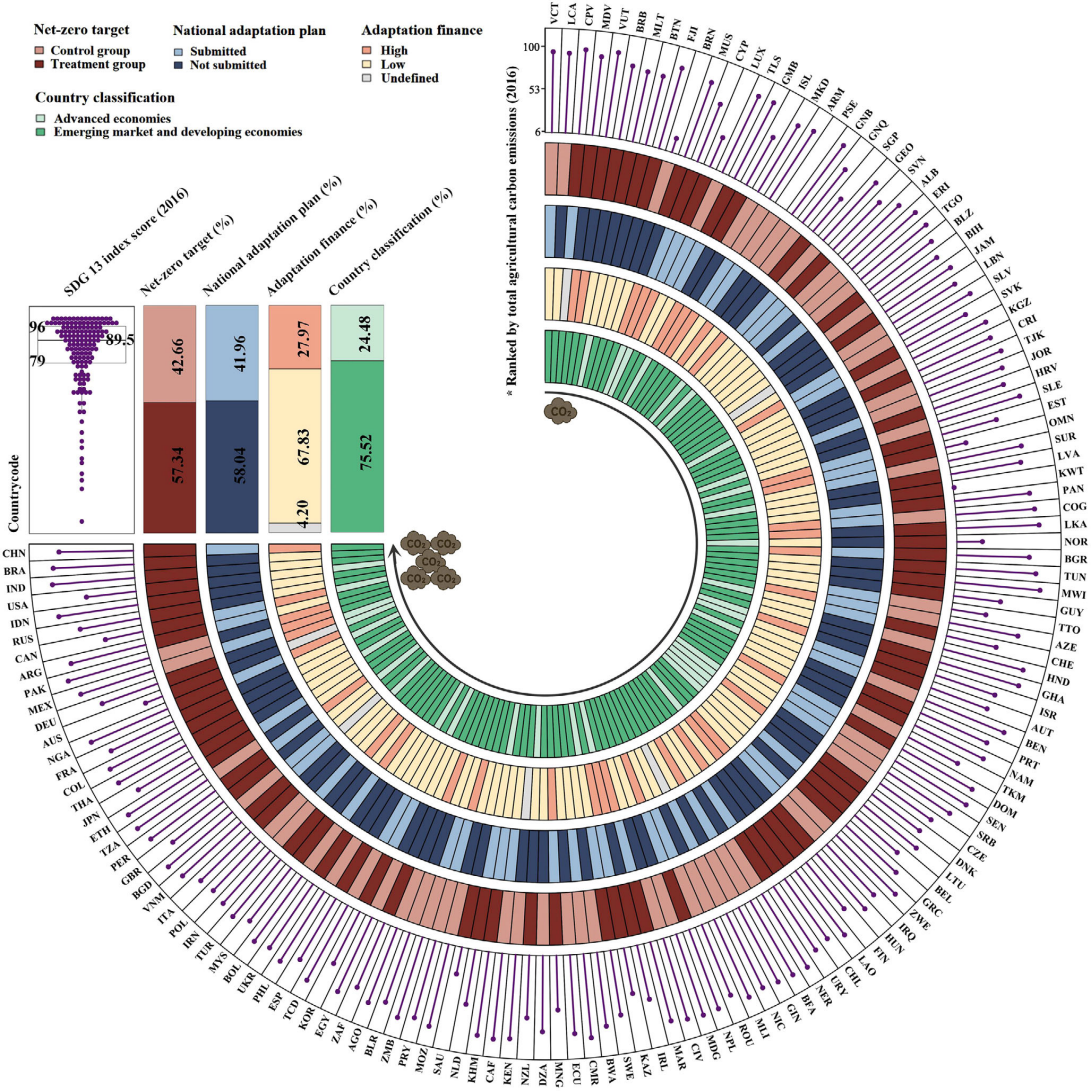
Characteristics of sample countries.

We attempted to construct a balanced panel dataset encompassing the broadest possible coverage of countries to ensure the reliability of our findings. *The Paris Agreement* places *Nationally Determined Contributions* (NDCs) at the center of global climate politics [20]. As of July 2025, 196 countries had submitted their NDCs, which constituted the ideal sample for our analysis. Owing to limitations in data availability, 53 countries were excluded because of missing data on agricultural output value or control variables. We firmly declared that sample exclusions are only attributable to exogenous factors such as statistical criteria and coverage of databases, which could be regarded as approximately random. Thus, there is no concern about sample selection bias. As depicted in Figure 1, the final sample comprises 143 countries, consisting of 35 advanced economies and 108 emerging market and developing economies. These economies demonstrated strong representativeness, since they collectively accounted for 75.81% of agricultural output value and 78.16% of total agricultural carbon emissions within FAO's statistical coverage. As noted by Bertrand et al. [21], extending the time window in DID estimation increases the risk of violating the parallel trends assumption. Notably, *the Paris Agreement* entered into force in 2016, serving as a natural starting point for our analysis, and most variables only updated through 2023. We eventually compiled a comprehensive dataset from multiple sources to empirically explore the agricultural economic impact of mitigation policy. This dataset encompasses information on each nation's policy implementation, agricultural production, climatic condition, and social governance.

#### 2.1.1 Core Explanatory Variable: Mitigation Policy

Currently, regarding researches on the impact of mitigation policy at the macro level, the mainstream approach is designing multiple mitigation policy scenarios for simulation within prediction models [22]. However, it is clearly unsuitable for exploring historical data. Even though the number of mitigation policies is vast, identifying one that is globally comparable remains hard. The high submission rate of NDCs renders them inappropriate for designing DID estimation, as the control groups would be insufficient. Fortunately, we identified net‐zero targets as a novel and viable perspective for macro‐level assessment. While net‐zero targets typically manifest as net‐zero carbon emissions, net‐zero emissions, and climate neutrality, we did not intentionally differentiate between these targets throughout our analysis and any aforementioned target constituted mitigation policy implementation [23]. Authoritative information regarding net‐zero targets was sourced from the Climate Watch Platform at https://www.climatewatchdata.org/net‐zero‐tracker.

**FIGURE 2:**
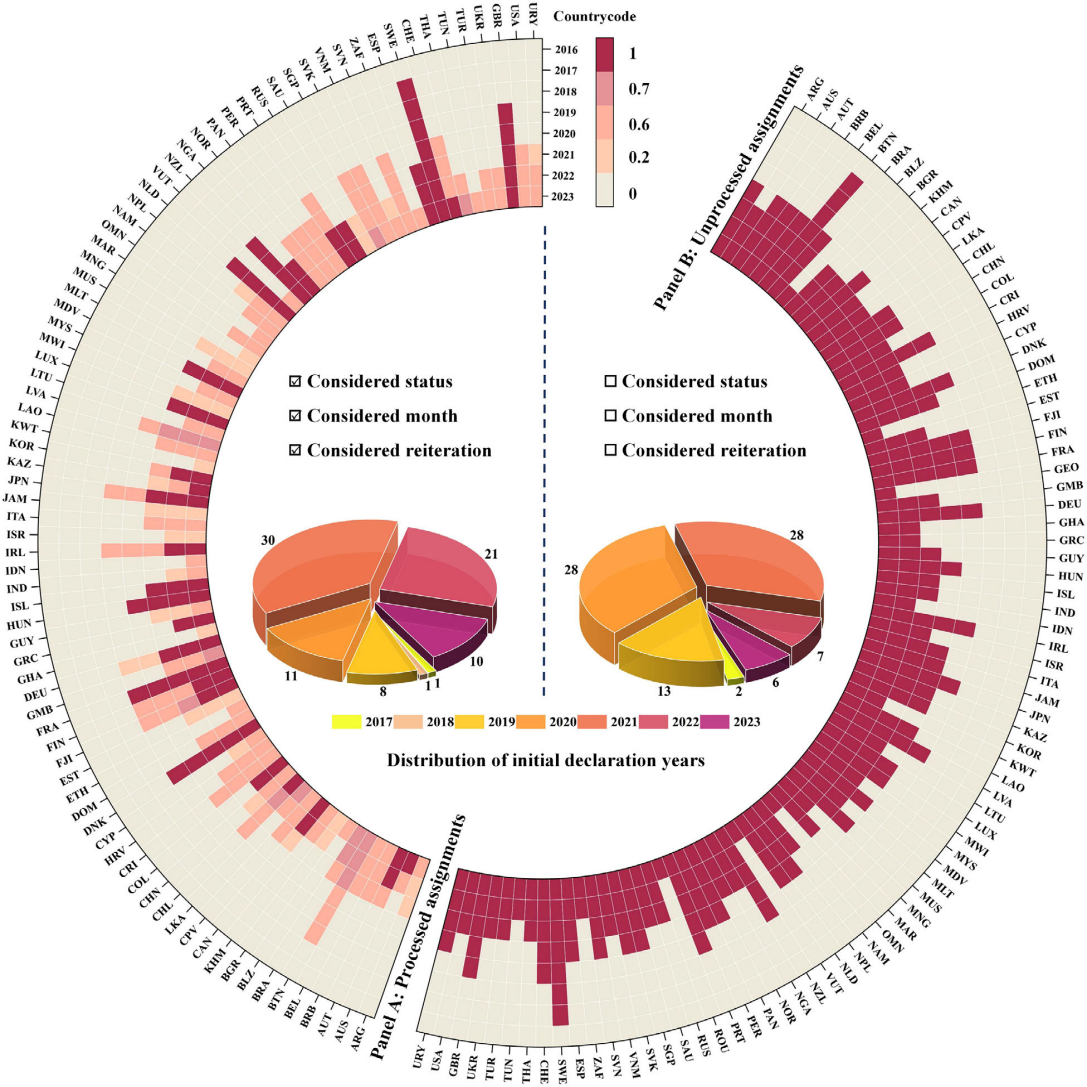
Characteristics of mitigation policy implementation.

Totally, 58.74% of sample countries communicated their net‐zero targets, with initial declarations concentrated in 2020 and 2021. Panel B of Figure 2 shows our core explanatory variable derived by unprocessed assignments (i.e., not considered status, month, and reiteration). However, distinct net‐zero target statuses reflect varying levels of policy stringency, which in turn lead to disparities in implementation intensity [24]. In accordance with net‐zero target statuses, our core explanatory variable was recoded: in political pledge (assigned 0.2), in policy document (assigned 0.6), and in law (assigned 1). When multiple statuses exist within a year, the maximum value is retained. Since value assignment by year would underestimate treatment effects [25], we strategically used value assignment by month. Specifically, initial declaration occurring from January to June was recorded in the current year, whereas those occurring from July to December was attributed to the next year. Additionally, net‐zero target status remains unchanged in subsequent years, but repeated declaration signifies heightened mitigation ambition and intensity. Accordingly, we added 0.1 for each reiteration, with the cumulative value not exceeding the next status. As described in Panel A of Figure 2, Georgia and Romania are excluded from the treatment group after processed assignments, with initial declarations concentrated in 2021 and followed by 2022.

#### 2.1.2 Dependent Variable and Control Variables

Agricultural and socioeconomic data were collected from the FAO Statistical Database (FAOSTAT) and World Development Indicators (WDIs). The dependent variable in this paper is agricultural output value ($1 billion), measured in constant 2014–2016 US dollars to ensure cross‐year and cross‐country comparability. Notably, *agriculture* is defined as generalized agriculture in accordance with the international standard. Aligning with the practice of Mamba and Ali [26], we include agricultural export value ($1 billion) and government effectiveness in the set of control variables. Theoretically, the nexus between agricultural export and agricultural economic growth can be articulated by the demand‐led growth theory. Empirical evidence indicates that government effectiveness enhances agricultural economic growth by improving the quality of public services [26]. Labor, land, and capital constitute the essential endowments underlying agricultural productivity [27]. Thus, labor input (%) and capital input (%) are likewise incorporated as control variables, measured by employment in agriculture as a share of total employment and gross fixed capital formation as a share of total GDP, respectively. Since agricultural sub‐sectors differ markedly in value‐added per unit, output structure (%) constitutes a critical determinant of agricultural output, measured by the proportion of cropping output value to total agricultural output value.

Indeed, agriculture is the most sensitive industry to climate change. Mittenzwei et al. [28] warned neglecting climatic factors in studies on agricultural production, land use, and welfare could result in tremendous analytical errors. Notably, current climate change is predominantly manifested as risk associated with climate warming [1]. Hence, we processed data on temperature change over land (°C) distributed by the National Aeronautics and Space Administration Goddard Institute for Space Studies (NASA‐GISS). Rare missing values were imputed using interpolation techniques to ensure data completeness. As reported in Table 1, the mean of temperature change over land is 1.476°C, showing that climate warming has been observed both regionally and globally. Additionally, we defined land input (one million of hectares) as the area of agricultural land use, and corresponding data are obtained in the FAOSTAT. Similarly, Table 1 reports diagnostic statistics, and all variance inflation factors (VIF) are below 10, confirming multicollinearity does not pose a significant concern in our model.

**TABLE 1:** Variables and descriptive statistics.

Variable	Mean	Std. dev.	Min	Median	Max	VIF
Agricultural output value	29.884	121.662	0.001	5.408	1439.300	
Mitigation policy	0.142	0.301	0	0	1	1.21
Agricultural export	10.728	22.601	0.000	1.796	191.738	1.73
Government effectiveness	0.058	0.917	−1.807	−0.051	2.317	2.70
Labor input	22.350	20.649	0.091	15.464	73.983	2.31
Capital input	4.849	4.494	0.060	3.712	38.977	1.38
Output structure	63.397	20.849	8.150	64.585	100.000	1.57
Temperature change	1.476	0.566	0.027	1.397	3.684	1.17
Land input	30.602	73.878	0.001	5.091	522.952	1.49

### 2.2 Model Specification

We conceptualize the declaration of net‐zero targets as a quasi‐natural experiment. Considering the staggered declaration timing across nations, we implement a multi‐period DID design to explore the potential impact of mitigation policy on agricultural output value, following an approach established by Beck et al. [29]. Nations that communicated net‐zero targets of any type are designated as the treatment group, whereas those without such declarations constitute the control group. Moreover, we identify the two‐way fixed effects (TWFE) model as the most appropriate specification for our analysis based on the Hausman test results. We successfully confirmed that heterogeneous treatment effects resulting from the staggered declaration timing were not severe in subsequent robustness checks, so stacked regression model was not adopted as our benchmark regression model. Equation (1) denotes the benchmark regression model.

(1) $$AOV_{it}=\beta_{0}+\beta_{1}MP_{it}+\beta X_{it}+\gamma_{i}+\mu_{t}+\varepsilon_{it}$$

where AOV_it_ denotes the agricultural output value of i country in t year. MP_it_ denotes a categorical variable for whether i country implemented mitigation policy in t year, taking one of five possible values: 0, 0.2, 0.6, 0.7, or 1. X_it_ denotes a set of control variables. γ_i_ and μ_t_ denotes country and year fixed effects, respectively. ε_it_ is a random disturbance term. β_0_ represents the constant term, while β_1_ and β represent the coefficients to be estimated.

The parallel trends assumption between treatment and control groups is fundamental to valid DID estimation. Drawing on established methodologies [30], we employ an event‐study approach to confirm whether agricultural output value maintained the parallel trends assumption prior to the declaration of net‐zero targets. Due to limited sample size in partial periods, which may lead to insufficient degrees of freedom when our control variables are incorporated, we collapse observations from periods earlier than 6 years before declarations into the ‐6th period and likewise observations from periods later than 3 years after declarations into the 3rd period in Panel A. The model is formally specified in Equation (2).

(2) $$AOV_{it}=\beta_{0}+\sum_{s=-6}^{3}\sigma_{s}MP_{is}+\beta X_{it}+\gamma_{i}+\mu_{t}+\varepsilon_{it}$$

where σ_s_ denotes the coefficient to be estimated and s denotes periods relative to initial declaration years, with the meanings of other symbols consistent with Equation (1).

## 3 Empirical Findings

### 3.1 Decarbonization at the Expense of Agricultural Output?

The benchmark regression results are presented in Table 2, where scenario 1 accounts for all control variables and scenario 2 does not account for any control variables. Guided by above assignment procedures, we sequentially estimated model (1) to model (4). As shown in model (4), the adverse impact of mitigation policy on agricultural output value passes the significance test at the 5% level. This conclusion is further corroborated by the first three models. Specifically, the mitigation policy is expected to diminish agricultural output value by 7.9%. Apparently, such phenomenon is not the original intention of mitigation policies, because decarbonization should not come at the expense of agricultural output. Combined with evidences from the industrial sector, we hold a view that a cost‐benefit analysis of mitigation policy should account for anticipated or realized losses. Certainly, our findings are not intended to undermine the significance of mitigation action in the agricultural sector. That is, our findings do not target any single specific measure. Our purpose is simply to demonstrate that there is no synergy between climate change mitigation and agricultural growth at present.

**TABLE 2:** Results of the benchmark regression model.

Variable	Agricultural output value					ACE
	Model (1)	Model (2)	Model (3)	Model (4)	Model (5)	Model (6)
Scenario 1: With controls						
Mitigation policy	−0.031**	−0.067**	−0.078**	−0.079**	−0.097**	−0.049***
	(0.014)	(0.030)	(0.039)	(0.039)	(0.048)	(0.011)
Output structure	0.019***	0.019***	0.019***	0.019***	0.019***	0.001
	(0.005)	(0.005)	(0.005)	(0.005)	(0.005)	(0.001)
Temperature change	−0.020*	−0.020*	−0.020*	−0.019*	−0.020*	−0.012**
	(0.011)	(0.011)	(0.011)	(0.011)	(0.012)	(0.006)
Adj R‐squared	0.9973	0.9974	0.9974	0.9974	0.9973	0.9993
Scenario 2: Without controls						
Mitigation policy	−0.037**	−0.073**	−0.087**	−0.088**	−0.105**	−0.052***
	(0.017)	(0.035)	(0.043)	(0.043)	(0.053)	(0.011)
Adj R‐squared	0.9968	0.9968	0.9968	0.9968	0.9967	0.9993
Considered status	No	YES	YES	YES	YES	YES
Considered month	No	No	YES	YES	YES	YES
Considered reiteration	No	No	No	YES	YES	YES
Country fixed	YES	YES	YES	YES	YES	YES
Year fixed	YES	YES	YES	YES	YES	YES
Observations	1144	1144	1144	1144	1001	1001

*Note*: *, **, and *** denote significance at the 10%, 5%, and 1% levels, respectively. Here, insignificant control variables are not reported for the sake of layout simplicity. The heteroskedasticity‐robust standard errors clustered at the individual level are displayed in parentheses. Notably, agricultural output value is log‐transformed throughout this paper to interpret the economic significance of treatment effects. Lastly, ACE stands for total agricultural carbon emissions, with a logarithmic transformation applied.

Indeed, our particular attention is paid to the comparison between the magnitude of output reduction and that of emission reduction. Thus, data on total agricultural carbon emissions were collected from the FAOSTAT. Nevertheless, these data are only available up to 2022. Results of models (5) and (6) indicate that the magnitude of emission reduction is nearly half that of output reduction. This harsh reality implies that decarbonization is not merely a sacrifice of agricultural output, but may lead to its destruction. Proportionate sacrifice still leaves room for improvement, whereas disproportionate destruction constitutes an unsustainable situation. Admittedly, this comparison is somewhat coarse, as it may overlook both the difficulty and the potential of agricultural carbon reduction. Nevertheless, its cautionary value in this context is substantial.

Although our primary focus lies in exploring the agricultural economic impact of mitigation policy, it must be acknowledged that traditional control variables exhibit considerable explanatory power. Temperature change in model (4) shows a significant negative impact on agricultural output value, aligning with prevailing scholarly views [31]. Climate warming will increase the uncertainty of agricultural production activities, thereby affecting their input‐output efficiency. Output structure determines the allocation of factors, thereby affecting overall productivity. Notably, not all control variables are expected to be statistically significant, as their inclusion primarily serves to erase omitted variable bias, not to test hypotheses about their own coefficients [32].

**FIGURE 3:**
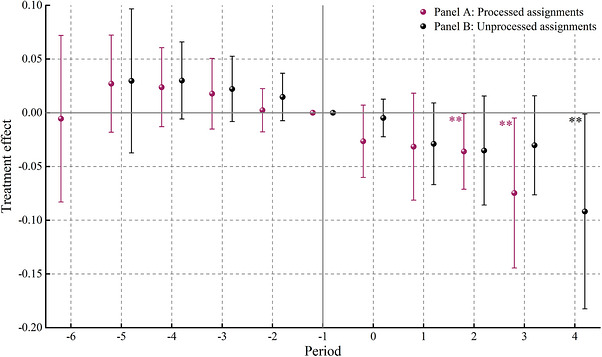
Parallel trend test. Notes: ** denotes significance at the 5% level. Similarly, we collapsed observations from periods earlier than 5 years before declarations into the ‐5th period and observations from periods later than 4 years after declarations into the 4th period in Panel B.

Figure 3 shows parallel trend test result for agricultural output value within 95% confidence interval, designating the ‐1st period as the reference period. Clearly, all treatment effects before declarations remain insignificant regardless of whether Panel A or Panel B is considered, indicating no essential disparities between our treatment and control groups in this paper. Conversely, partial treatment effects after declarations are significantly negative and exhibit a substantially increasing tendency, demonstrating lower agricultural output value in the treatment group compared to the control group. Moreover, the adverse impact of mitigation policy on agricultural output value displays a notable time‐lagged pattern, primarily attributed to transitions from official declaration to actual action requires substantial amount of time, so these observable impacts of net‐zero targets manifest only after a certain duration. Lastly, as mitigation policy expands both geographically and typologically over time, its adverse impact naturally manifest cumulative properties. Notably, results of Panel A are remarkably superior to that of Panel B, indicating that our tailored processing of assignments is warranted. Overall, our benchmark regression model satisfies the parallel trends assumption.

### 3.2 Robustness Check

However, potential biases may arise from factors such as sample selection and variable specification. Hence, we conducted corresponding robustness checks to address these concerns, and all results as depicted in Table 3. Regarding sample selection, we excluded 11 countries (including Luxembourg, Kuwait, Germany, etc.) where agricultural production constitutes an exceedingly small share (i.e., below 1%) of economic development in model (1). The primary rationale for this action lies in the fact that data on agricultural output value in such countries are often affected by the low base effect, resulting in relatively poor data quality. An alternative technique to address the distortion caused by outliers is winsorization. We applied two‐sided winsorization to each continuous variable at the 1% level in model (2). Results show that the absence of outliers that significantly affect the conclusion, indicating a certain degree of robustness in our benchmark regression results. Regarding variable specification, a handful of scholars might question the reliability of using net‐zero targets as a proxy for mitigation policy. Our current core explanatory variable is discrete, and we attempted to replace it with a continuous variable. Specifically, Wu et al. [2] developed the Global Climate Change Mitigation Policy Dataset (GCCMPD) by employing a semi‐supervised hybrid machine learning approach. We replaced a single policy shock with a broad policy shock by employing the number of mitigation policies as a proxy variable for estimation in model (3). Clearly, the result based on given continuous variable are fundamentally consistent with those derived from the DID estimation.

**TABLE 3:** Results of robustness check.

Variable	Agricultural output value					
	Model (1)	Model (2)	Model (3)	Model (4)	Model (5)	Model (6)
Mitigation policy	−0.091**	−0.039**	−0.073*	−0.072***	−0.079**	−0.166*
	(0.045)	(0.015)	(0.041)	(0.026)	(0.039)	(0.098)
Control variables	Controlled	Controlled	Controlled	Controlled	Controlled	Controlled
Country fixed	YES	YES	YES	YES	YES	YES
Year fixed	YES	YES	YES	YES	YES	YES
Adj R‐squared	0.9971	0.9983	0.9988	0.9967	0.9974	0.9982
Observations	1056	1144	858	4072	1144	952

*Note*: *, **, and *** denote significance at the 10%, 5%, and 1% levels, respectively. As the GCCMPD is updated only through 2021, model (3) is estimated based on 858 observations. The heteroskedasticity‐robust standard errors clustered at the individual level are displayed in parentheses. Notably, each treated‐group observation is matched to a control‐group observation in the stacked regression estimation. Since some control‐group observations may serve as matches for multiple treated‐group observations, the total number of observations in model (4) will exceed that of our original data. Indeed, controlling individually for extreme weather events or natural disasters produced identical results in model (5). Owing to missing data on energy structure, our endogeneity analysis in model (6) is equally conducted on a reduced sample. The Adjusted R‐squared in model (6) refers specifically to the Centered R‐squared.

Moreover, our DID estimation constitutes weighted average of multiple canonical DID estimators under staggered treatment. Crucially, discrepancy in treatment timing causes earlier‐treated group to serve as the control group for later‐treated group, but TWFE estimation regularly cannot handle these heterogeneous treatment effects [25]. Greater weighting of such control groups will amplify bias caused by cross‐period contamination. Drawing on the diagnostic approach of Goodman‐Bacon [33], decomposition results indicate that the time‐varying treatment group assigns 93.16% of its control‐group weight to never‐treated units. Accordingly, we concluded that the bias attributable to heterogeneous treatment effects is minimal. Even with negligible bias, we constructed a stacked regression estimation in model (4) of Table 3 following the methodology of Cengiz et al. [34]. The estimated coefficient for mitigation policy in model (4) is calculated based on the coefficients for the current period and the subsequent six periods. Clearly, after erasing the interference from bad control groups, our main conclusion remains valid.

Indeed, we are concerned that the observed output reduction could stem from other unaccounted confounding factors. The Task Force on Climate‐Related Financial Disclosure characterizes climate physical risk as uncertainties stemming from climate factors such as extreme weather events, natural disasters, and global warming [35]. Nevertheless, the agricultural economic impact of global warming has been demonstrated to be less immediate and pronounced than those of extreme weather events and natural disasters. The losses inflicted by both can be considered catastrophic. Drawing on the EM‐DAT database, extreme weather events are categorized into four primary types: drought, extreme temperature, flood, and storm. Natural disasters primarily encompass biological, hydrological, climatological, geophysical, meteorological, and extra‐terrestrial disasters. Consistent with the methodology of Wen et al. [36], we employed the total number of people affected as proxy variables. As indicated by model (5) of Table 3, controlling for the aforementioned factors does not alter our benchmark regression result in model (4) of Table 2.

**FIGURE 4:**
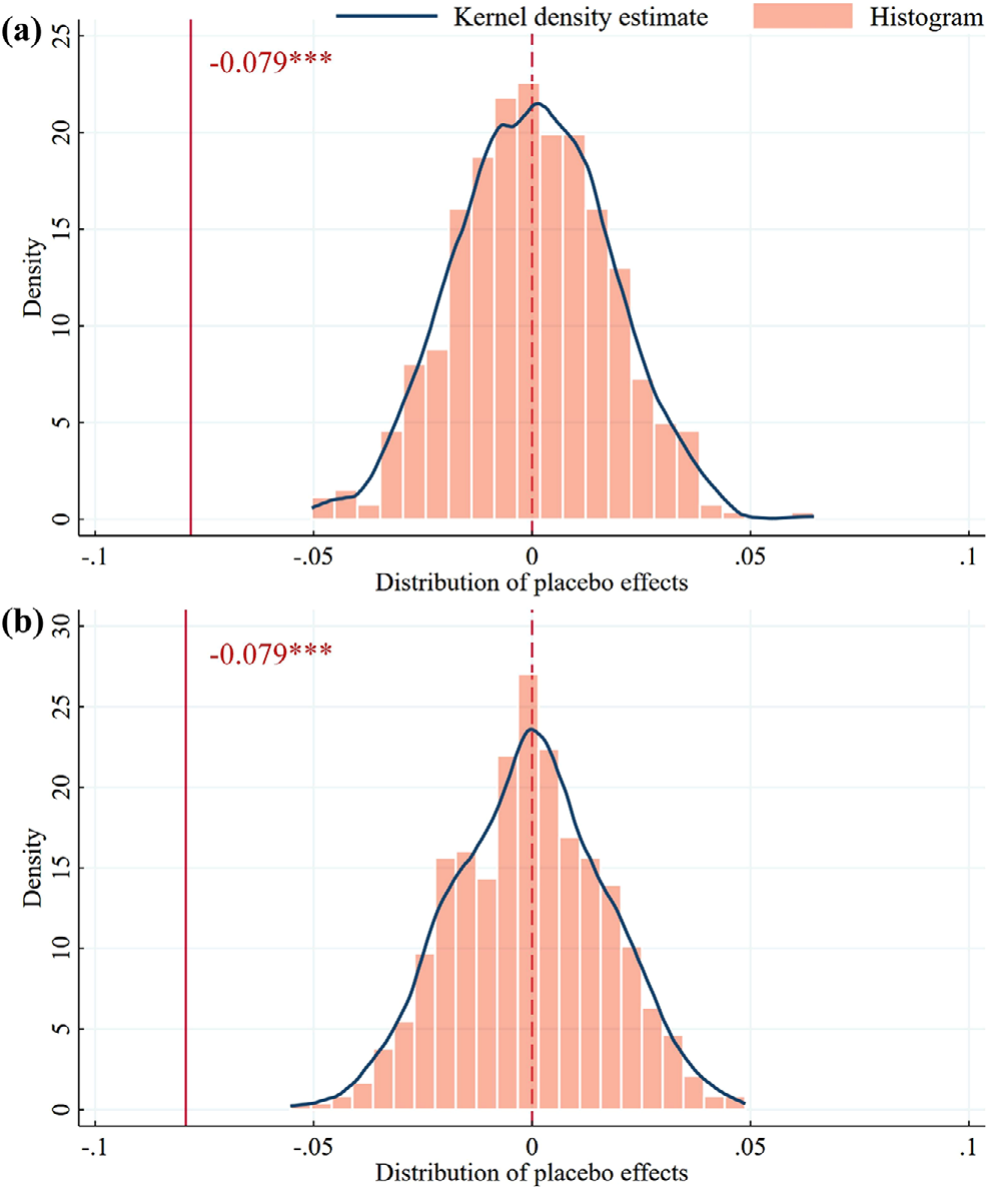
Restricted (a) and unrestricted (b) mixed placebo tests.

While foregoing procedures exclude those factors that can be easily identified, more unobservable and unquantifiable factors will be addressed via placebo tests. Specifically, the placebo test typically involves temporal and spatial dimensions, and the mixed placebo test randomly assigns a subset of nations to the treatment group with randomized declaration timestamps. Meanwhile, our benchmark regression model will be re‐estimated to obtain new estimated coefficients for mitigation policy. Persistent significance in these falsified coefficients indicates bias in benchmark regression results, thereby rejecting the potential causal interpretation in favor of the actual coincidental association. Following 500 replications, whether or not to restrict the original group structure, our true treatment effect resides in the left tail of the placebo distribution, as depicted in Figure 4. Hence, all procedures lend stronger empirical supports to our core finding.

We attempted to use the Bartik instrumental variable constructed based on the share‐shift method to address endogeneity concerns. The required instrumental variable can be constructed by combining particular policy shocks with the corresponding unit‐level shares, calculated as a weighted average, allowing flexible implementation across contexts [37]. The Bartik instrumental variable is obtained by multiplying fossil fuel energy consumption (% of total) in 2000 with the U.S. climate policy uncertainty index in this paper. The historical energy structure is a critical consideration in the design of mitigation policy. Using the U.S. climate policy uncertainty index as a proxy for global conditions constitutes a common practice in the literature [38]. Additionally, these two indicators do not directly affect agricultural output value. Thus, our Bartik instrumental variable meets both the relevance and exogeneity conditions required for valid identification. Relevant data were collected from the WDIs and https://www.policyuncertainty.com/index.html. Results indicate that the Kleibergen‐Paap rk LM statistics satisfy conventional significance thresholds, rejecting the null hypothesis of under‐identification, and the Kleibergen‐Paap rk Wald F statistics exceed the 10% critical values, confirming the absence of weak instrument problems. Clearly, as shown in model (6) of Table 3, our main finding remains valid after correcting for endogeneity concerns.

Lastly, since the declaration of net‐zero targets does not constitute a strict quasi‐natural experiment, many scholars may advocate for addressing this concern through propensity score matching [39]. Propensity score matching, as a prevalent data preprocessing technique, aligns the observable characteristics of the treatment and control groups as closely as possible. Notably, net‐zero targets are initially intended to reduce carbon emissions, with their ultimate goal being to advance climate‐related sustainable development. Hence, countries with higher agricultural carbon emissions or lower SDG13 index scores are more likely to be included (or included earlier) in the treatment group. However, as described in Figure 1, these two indicators are not necessarily linked to the declaration of net‐zero targets. Taken together, these analyses suggest no compelling evidence of sample self‐selection bias in our model.

### 3.3 Attributed to Stagnant Technology

We seek to uncover why mitigation policy impedes agricultural growth through mechanism analysis. Indeed, agricultural growth is heavily reliant on agricultural material inputs. For instance, Oerke [40] believed that pesticide use enabled agricultural producers to modify production systems and to increase the agricultural productivity without sustaining the higher losses likely to occur from an increased vulnerability to pest infestation. Pesticides are not exclusive to crop production, and they also have crucial application potential in the livestock sector, particularly in the control of parasitic infestation. Nevertheless, carbon emissions from pesticide use constitute a non‐negligible threat to global climate [41]. Naturally, curbing pesticide use and other agricultural material inputs has become a vital content of mitigation policy. Empirical evidences for this insight are shown in models (1) and (2) of Table 4. These results represent a combination of proactive motivation and compelled response. On the one hand, since agriculture is particularly exposed to escalating climate risk, agricultural producers respond with heightened urgency and sense of responsibility in climate governance [32]. On the other hand, since mitigation policy had been considered for inclusion in the national legislative agenda in some countries, agricultural producers are compelled to take action.

**TABLE 4:** Results of mechanism analysis.

Variable	Pesticide use		Mitigation patent	
	Model (1)	Model (2)	Model (3)	Model (4)
Mitigation policy	−0.174***	−0.174***	−3.454*	−3.870**
	(0.066)	(0.063)	(1.938)	(1.961)
Control variables	Controlled	Uncontrolled	Controlled	Uncontrolled
Country fixed	YES	YES	YES	YES
Year fixed	YES	YES	YES	YES
Adj R‐squared	0.9881	0.9881	0.8978	0.8956
Observations	1136	1136	686	686

*Note*: *, **, and *** denote significance at the 10%, 5%, and 1% levels, respectively. Complete data for mechanism variables are unavailable. For instance, pesticide use data obtained from the FAOSTAT are missing for Singapore. Verification indicates that re‐estimating Equation (1) employing the remaining observations produces results consistent with our benchmark regression. The heteroskedasticity‐robust standard errors clustered at the individual level are displayed in parentheses.

Based on endogenous growth theory, long‐term economic growth can be achieved independently of external inputs. Pursuing this sustainable status largely depends on technological progress. Hence, we employed the unbalanced panel data regarding mitigation patent from the OECD Data Explorer as another mechanism variable in models (3) and (4) of Table 4. Results reveal that mitigation policy exerts a pronounced negative impact on mitigation technology. Due to limitations in data availability, we cannot collect an adequate number of observations regarding mitigation patent in agriculture. Given its narrow profit margins, technological progress in agriculture has typically lagged behind other sectors [42]. It is reasonable to assume that, given the overall stagnation in mitigation technology, the agricultural sector is unlikely to have experienced notable advancement. Clearly, grounded in the principle of the Cobb–Douglas production function, a marked reduction in agricultural material inputs without corresponding technological progress inevitably translates into lower agricultural output value. Agricultural producers may perceive this as highly irresponsible, as governments likewise bear the obligation to foster and supervise the development of mitigation technology [42]. Here, we pessimistically found that the Porter hypothesis did not hold in this context.

### 3.4 Compensation from Adaptation Policy

The Gaia hypothesis contends that life is both a shaper of the Earth's environment and an organismal system that adapts to it [43]. Hence, mitigation and adaptation constitute two primary strategies for addressing escalating climate change. Specifically, mitigation focuses on intergenerational justice, while adaptation prioritizes immediate survival. *The Cancun Agreement* formally acknowledged adaptation as a core component of the international climate policy agenda, while *the Paris Agreement* explicitly articulated a global adaptation purpose aimed at enhancing adaptation capacity, strengthening resilience, and reducing vulnerability to climate change. With growing recognition of the significance of adaptation policy, the agricultural sector exhibits the highest levels of both coverage and adoption of adaptation measures, typically including cultivation of heat‐resilient seed varieties, alteration in grazing schedules, and so on [44]. The agricultural economic impact of adaptation policy has been widely documented in studies at both micro and macro scales. For instance, Nam et al. [45] reported that Vietnamese rice farmers who switched rice varieties achieved a 24.5% increase in yield and a 73% increase in additional profit. Kahsay and Hansen [46] pointed out that adaptation policy capable of neutralizing the impact of intensified precipitation variability during the main season could increase the net production index by no less than 0.17% to 0.29% in East Africa. However, can adaptation policy fully compensate for the losses in agricultural output inflicted by mitigation policy? We examined this proposition through heterogeneity analysis.

Countries were encouraged to develop National Adaptation Plans (NAPs) to facilitate planning and communication on national and subnational adaptation priorities [47]. As of July 2025, a total of 74 countries worldwide had submitted their NAPs. Hence, we classified 143 sample countries into high‐ and low‐adaptation policy countries based on submission status. Moreover, we collected each country's total adaptation finance within our study period from the international multilateral public climate finance database provided by Fan et al. [48] and reclassified the sample countries based on the mean value. Both classification methods have inherent limitations, and their joint application is crucial to thoroughly explore the compensatory effect of adaptation policy on mitigation policy. Lastly, the distribution of subgroups is illustrated in Figure 1. As shown by Table 5, the adverse impact of mitigation policy on agricultural output value is observed only in low‐adaptation policy countries, confirming our foregoing proposition. These findings are encouraging, suggesting an intrinsic coherence among various types of climate policies. More importantly, they serve as a cautionary notice that narrowly criticizing climate policy from a single view is inadvisable.

**TABLE 5:** Results of heterogeneity analysis.

Variable	Agricultural output value				
	Classified by NAPs		Classified by adaptation finance		
	Model (1)	Model (2)	Model (3)	Model (4)
Mitigation policy	−0.008	−0.094**	−0.025	−0.107**
	(0.029)	(0.045)	(0.038)	(0.052)
Adaptation policy	High	Low	High	Low
Control variables	Controlled	Controlled	Controlled	Controlled
Country fixed	YES	YES	YES	YES
Year fixed	YES	YES	YES	YES
Adj R‐squared	0.9980	0.9973	0.9982	0.9965
Observations	480	664	320	776

*Note*: ** denotes significance at the 5% level. The heteroskedasticity‐robust standard errors clustered at the individual level are displayed in parentheses. Authoritative information regarding NAPs is publicly accessible at https://napcentral.org/about‐naps. Unfortunately, adaptation finance data for eight countries are unavailable, and thus these countries are undefined in models (3) and (4).

### 3.5 Social Impact of Mitigation Policy

As previously mentioned, the effectiveness of mitigation policy encompasses at least environmental, economic, and social dimensions. Here, our focus shifts to the social dimension. Far beyond a mere economic sector, agriculture deeply embedded within social systems. Without doubt, the agricultural economic impact of mitigation policy inevitably triggers a series of interconnected social responses. Treating agricultural output value as a mechanism variable, we sought to conduct a more in‐depth sociological analysis. Notably, this approach has been proven as practical through peer review. For instance, Lai et al. [20] also treated agricultural output value as the mechanism variable to assess how adaptation policy influence progress toward No Poverty.

Reducing poverty has stood at the forefront of international debates since the 1970s. Considering that three‐quarters of the global poor depend on agriculture for their livelihoods, agricultural growth is more effective at reducing poverty than growth in other sectors. Moreover, the World Bank [49] emphasized the advancement of the agricultural sector is determinant for many developing countries to escape poverty traps. Conversely, does the reduction in agricultural output value observed in Section 3.1 exacerbate poverty? To verify this proposition, we examined whether mitigation policy (SDG13) affects progress toward No Poverty (SDG1) in models (1) and (2) of Table 6. Notably, our empirical strategy has been recognized in the existing literature as both feasible and important. For instance, Wu et al. [50] maintained that the SDGs interact dynamically and identified decoupling‐recoupling dynamics among various SDGs via relational networks. Results indicate that mitigation policy exhibits a statistically significant negative impact on SDG1 index score. The aforementioned proposition is confirmed, as losses of critical livelihoods do intensify existing poverty conditions. The rational choice theory implies that as mitigation policy focuses on intergenerational justice, its priority dramatically diminishes when confronted with pressing survival challenges, resulting in individuals' withdrawal from mitigation strategies. Put differently, the public foundation for climate action may be weakened under this scenario.

**TABLE 6:** Results of sociological analysis.

Variable	SDG1 index score		SDG index score		
	Model (1)	Model (2)	Model (3)	Model (4)
Mitigation policy	−2.000***	−2.353***	−0.301***	−0.392***
	(0.653)	(0.699)	(0.133)	(0.139)
Control variables	Controlled	Uncontrolled	Controlled	Uncontrolled
Country fixed	YES	YES	YES	YES
Year fixed	YES	YES	YES	YES
Adj R‐squared	0.9905	0.9899	0.9962	0.9956
Observations	1016	1016	1136	1136

*Note*: *** denotes significance at the 1% level. The heteroskedasticity‐robust standard errors clustered at the individual level are displayed in parentheses. Data for SDG1 and SDG index score can be collected from https://dashboards.sdgindex.org/explorer. Notably, these data are unavailable for a limited number of countries. Verification indicates that re‐estimating Equation (1) employing the remaining observations produces results consistent with our benchmark regression.

## 4 Further Discussion

**FIGURE 5:**
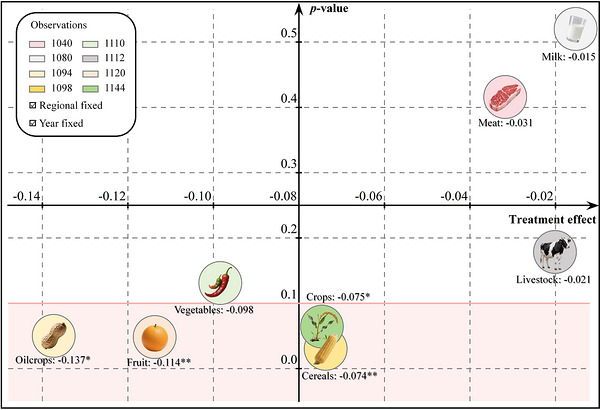
Results of agricultural output value by components. Notes: * and ** denote significance at the 10% and 5% levels, respectively. Owing to limitations in data availability, partial models are based on unbalanced panel data and components with excessive missing values are excluded from estimations. Moreover, oilcrops, fruit, vegetables, and cereals refer to primary products and meat specifically denotes indigenous meat. The inclusion of all control variables is achieved.

Agriculture is an inherently composite sector, each component characterized by distinct production technologies, input structures, and sensitivity to external shocks. Exploring agricultural output value by components may offer more valuable insights. Based on the FAOSTAT, we classified agricultural output value into two categories including crops and livestock, and further into six subcategories including oilcrops, fruit, vegetables, cereals, meat, and milk. The visualization of estimated results is reported in Figure 5. Clearly, unlike livestock, the output value of crops is adversely affected by mitigation policy, with oilcrops being the most impacted, showing an estimated reduction of 13.7%. Building on the conclusion from Section 3.3, such heterogeneity is attributed to low input intensity in the livestock sector, where the dominant sources of carbon emissions are enteric fermentation and manure management [51]. Indeed, this disparity is widely acknowledged in the existing literature [52]. Accordingly, subsequent mitigation efforts should place particular emphasis on alleviating output reduction of crops.

As renowned author Douglas Adams once quipped, *‘'We are stuck with technology when what we really want is just stuff that works'’*. The supply and demand for technology appear to operate in a dynamic cycle. Indeed, an urgent demand for mitigation technology among agricultural producers is undeniable. But why supply fails to keep pace? As indicated by models (1) and (2) of Table 7, mitigation policy does not produce a statistically significant expansion in mitigation finance, which is arguably a deterministic factor constraining supply for mitigation technology [53]. In this context, our policy implication goes beyond advocating mitigation technological innovation in agriculture. More effective action requires addressing root causes by leveraging both economic and reputational incentives to attract greater social capital into mitigation financing. Meanwhile, Tol [54] inferred that mitigation policy would exacerbate fiscal expenditure pressures and prompt a reallocation of budgetary structure. Hence, environmental protection expenditure may crowd out certain allocations to agriculture, thereby hindering agricultural growth. Notably, as indicated by models (3) and (4) of Table 7, results regarding fiscal expenditure on agriculture as an additional mechanism variable are insignificant, indicating above proposition can be dismissed. Certainly, it is also permissible if you interpret fiscal expenditure on agriculture as one of the funding sources for mitigation technological innovation in agriculture.

**TABLE 7:** Results of further mechanism analysis.

Variable	Mitigation finance		Fiscal expenditure on agriculture	
	Model (1)	Model (2)	Model (3)	Model (4)
Mitigation policy	0.169	0.179	0.021	0.047
	(0.119)	(0.121)	(0.192)	(0.178)
Control variables	Controlled	Uncontrolled	Controlled	Uncontrolled
Country fixed	YES	YES	YES	YES
Year fixed	YES	YES	YES	YES
Adj R‐squared	0.4673	0.4694	0.8958	0.8963
Observations	1096	1096	966	966

*Note*: The heteroskedasticity‐robust standard errors clustered at the individual level are displayed in parentheses. Data for additional mechanism variables were collected from the international multilateral public climate finance database and FAOSTAT. Owing to limitations in data availability, partial models are based on unbalanced panel data. Verification indicates that re‐estimating Equation (1) employing the remaining observations produces results consistent with our benchmark regression.

Undoubtedly, the finding that adaptation policy can largely compensate for output reduction induced by mitigation policy is inspiring. This finding underscores the imperative of balancing mitigation and adaptation policies in future practice, as these two strategies are in competition, with adaptation occupying a subordinate position. Given finite climate governance resources, governments typically prioritize based on their perceived values, and mitigation policy tied to international obligation and reputation naturally receive higher precedence [55]. Moreover, this imbalance may also be historically rooted. The first scientific paper linking fossil fuel combustion to global warming was published in 1896. Since then, international academic and political debates regarding emission reduction responsibility have intensified [56]. Indeed, we are not the first to advance this initiative. McCarl [57] has forcefully advocated for the necessity of adaptation and mitigation in collectively shaping sustainable agriculture.

We inferred that losses of critical livelihoods significantly reduce SDG1 index score in Section 3.5. Although this insight has received empirical support from numerous scholars [58], we must acknowledge that our empirical strategy lacks rigor. This is because we cannot rule out the possibility that the observed social impact of mitigation policy operates through other mechanisms. Our insight focuses on income, while a perspective of expenditure remains unexplored. Specifically, poverty tends to rise when mitigation policy exerts regressive distributional impacts by increasing the cost of essential goods such as food, energy, or mobility [7]. We will further explore this limitation through dedicated thematic analyses in the future. However, such potential bias does not fundamentally invalidate the policy implication of this insight. In other words, establishing equitable climate redistribution rules remains a critical choice for governments [59].

Viewed through a long‐term lens, all endeavors we undertake today are ultimately directed toward achieving a sustainable future. Since mitigation policy exhibits a pronounced adverse impact on SDG1 index score, it is reasonable to concern that similar impact may extend to SDG index score. Models (3) and (4) of Table 6 report all estimated results. It is discouraging to observe that mitigation policy impedes progress toward sustainable development. Considering that mitigation efforts are core elements of SDG13, such phenomenon can be described as being “*penny wise, pound foolish*.” A plausible explanation is that mitigation policy was originally formulated without considering all aspects of sustainability. The UNDESA announced that only 33% of nations have established partial linkages between their NDCs and SDGs [20]. Clarifying the underlying mechanisms of this phenomenon constitutes a critical part of our research agenda going forward.

Interestingly, do our macro‐level results align with the perceptions of micro‐level actors? In 2021, Mittenzwei et al. [60] conducted a large population survey in Norway on the perceived impacts of mitigation policy on agriculture and rural areas. Results reveal that 71% of respondents believed that mitigation policy must not lead to reduced food production. Such finding contradicts our core conclusion and we offer a plausible explanation. Building on bounded rationality theory, agricultural producers are hard to accurately attribute the causes of output reduction. Additionally, agricultural producers in Norway assigned a much higher priority to climate policy than to other policy domains [60], and they are less likely to attributed it to mitigation policy even when production declines. We further argued that such perceptual bias or lag may be linked to the level of economic development. To explore this supposition, our team conducted a comparable field survey in a developing country, namely China. It was found that 73.8% of respondents are of the view that climate change mitigation measures decrease agricultural production. We posit that the perceptions of climate policies among agricultural producers in developing countries are likely more grounded in reality. Hence, future policymaking must give greater weight to their voices. That said, a more rigorous examination of this argument warrants future investigation.

## 5 Conclusion

How to craft a coherent and proactive framework to manage climate mitigation has become a global governance imperative amid intensifying climate challenges. Taking net‐zero targets as an example, we empirically explored the effectiveness of mitigation policy in this paper, with a particular focus on its agricultural economic impact. We must acknowledge a disproportionate relationship between the scale of emission reduction and output reduction under current mitigation efforts. This finding renders its economic effectiveness untenable in the agricultural sector. A similar conclusion emerges in the social dimension, as exemplified by rural poverty. Overall, the assessment of its effectiveness must move beyond a narrow focus on emission reduction outcomes, as decarbonization cannot come at the expense of agricultural output and poor agricultural producers' welfare. Meanwhile, we found that observed agricultural economic losses mainly stem from stagnant technological progress failing to compensate for declining material inputs. However, effective adaptation measures show potential to deliver such compensatory effect. More critically, vulnerable agricultural systems are struggling to withstand the dual pressures of climate change and climate mitigation. We must refine mitigation policy guidelines with unwavering courage and determination to create a legacy of change that will stand the test of time.

## Ethics Approval Statement

The authors have nothing to report.

## Conflicts of Interest

The authors declare no conflicts of interest.

## Data Availability

The data that support the findings of this study are available from the corresponding author upon reasonable request.
